# The impact of bidirectional emotional support on social isolation in older adults: the mediating role of depression and the moderating role of activities of daily living

**DOI:** 10.3389/fpubh.2025.1678246

**Published:** 2025-09-30

**Authors:** Chunhui Yang, Zhongsu Shi, Junnan Song, Huan Liu, Lin Zheng, Annuo Liu

**Affiliations:** School of Nursing, Anhui Medical University, Hefei, Anhui, China

**Keywords:** bidirectional emotional support, depression, social isolation, ADL, older adults

## Abstract

**Objective:**

To investigate the impact of bidirectional emotional support on social isolation among community-dwelling older adults and the underlying mechanisms of this effect.

**Methods:**

In this study, 1,136 community-dwelling older adults were recruited from Hefei City, China in 2022 using a stratified random sampling method. Data were collected using the General Information Questionnaire, the Intergenerational Support Scale, the Social Network Scale, the Geriatric Depression Scale, and the Activities of Daily Living (ADL) Scale. Correlation analyses, as well as mediation and moderation tests, were conducted to examine the relationship between bidirectional emotional support and social isolation.

**Results:**

Bidirectional emotional support significantly predicted (β = 0.213, *P* < 0.01) social isolation in older adults. Depression partially mediated the relationship between bidirectional emotional support and social isolation, with an indirect effect of 0.066, representing 23.66% of the total effect. ADL substantially moderated (β = 0.068, *P* < 0.01) the impact of bidirectional emotional support on depression.

**Conclusion:**

Bidirectional emotional support is a key modifiable factor influencing social isolation. Its impact is partly mediated by depressive symptoms and is also moderated by ADL. These findings suggest that “bidirectional emotional support” should be incorporated into the National Essential Public Health Services Program. Depression screening should be conducted during primary care visits, and emotional support should be provided in a tiered manner based on activities of daily living capabilities to alleviate social isolation among older adults.

## 1 Introduction

Social isolation refers to a condition in which individuals, either actively or passively, lack interactions and contact with others and society, as well as meaningful social relationships and support, leading to the narrowing or eventual disappearance of their social networks (1). The global prevalence of social isolation among community-dwelling older adults is 26% (2), with rates of 24% in the United States and 31.5% in Japan (3, 4). In China, the prevalence exceeds the global average, reaching 31.2% (5). Social isolation has emerged as a major public health concern, particularly in aging societies, due to its association with physical and mental health decline.

Social isolation has been linked to an increased risk of developing chronic diseases (6). Data from a 2020 U.S. Department of Health survey indicated that older adults experiencing social isolation face an increased risk of depression (31%), coronary heart disease (29%), stroke (32%), dementia (50%), hospital readmissions (26%), and suicide (18%) (7, 8). A meta-analysis of cohort and case-control studies involving over 2.3 million participants found that individuals who experience social isolation or loneliness are at a higher risk for both physical and mental health problems (9). Moreover, social isolation is associated with increased utilization of health services, including visits to physicians, emergency room admissions, and hospital readmissions (10). In an aging society, social isolation has emerged as a critical public health issue requiring thorough investigation and intervention.

In this context, many scholars have investigated the factors and mechanisms that contribute to alleviating social isolation in the older adults (11–13). Some research indicates that the wellbeing of older adults depends more on the quality than the quantity of social support, with those receiving higher levels of support being less prone to social isolation (14, 15). Intergenerational support plays a pivotal role within social support systems, alleviating loneliness and enhancing social engagement by providing instrumental, financial, and emotional assistance (16). Furthermore, research suggests that bidirectional emotional support is more effective than one-way assistance in strengthening emotional bonds (17). Therefore, further investigation is needed into the buffering effect of bidirectional emotional support in alleviating social isolation and its underlying mechanisms.

Bidirectional emotional support refers to the intergenerational relationship between the older adults and their children or grandchildren, wherein they exchange emotional care, understanding, companionship, and respect. This form of support does not emphasize a unidirectional exchange but rather highlights the bidirectional flow of emotions between the two parties, fostering a virtuous cycle of emotional connection within the family. Currently, the majority of studies focus on the influence of holistic or unidirectional intergenerational support on the mental health of older adults (18, 19). Some scholars have started exploring the role of bidirectional emotional support. Research suggests that bidirectional emotional support is particularly effective in alleviating negative emotions among older adults (20). The emotional bonds and comfort it fosters surpass those offered by other forms of support (21). Additionally, older adults who receive only support may develop feelings of dependency and guilt, which can undermine some of the benefits (22). However, most of these studies have focused on the role of bidirectional emotional support in enhancing the wellbeing and mental health of older adults, without specifically examining its impact on social isolation and the underlying mechanisms.

To further explore the potential mechanism of the impact of bidirectional emotional support on social isolation, scholars have conducted research from both aspects of mental health and physical functioning in older adults (23). Research indicates that bidirectional emotional support alleviates adverse psychological states and reduces depressive symptoms in older adults under stress (24–26). Emotional support within intergenerational relationships serves as a significant protective factor against depression, with an impact that often surpasses even daily care and financial support (27, 28). Furthermore, Santini et al. (29) suggest that depressive symptoms impair social networks, acting as a significant risk factor for social isolation (30). Intergenerational emotional support is linked to reduced levels of social isolation and functions as a critical determinant of wellbeing among older adults in South Asia (31).

**Figure 1:**
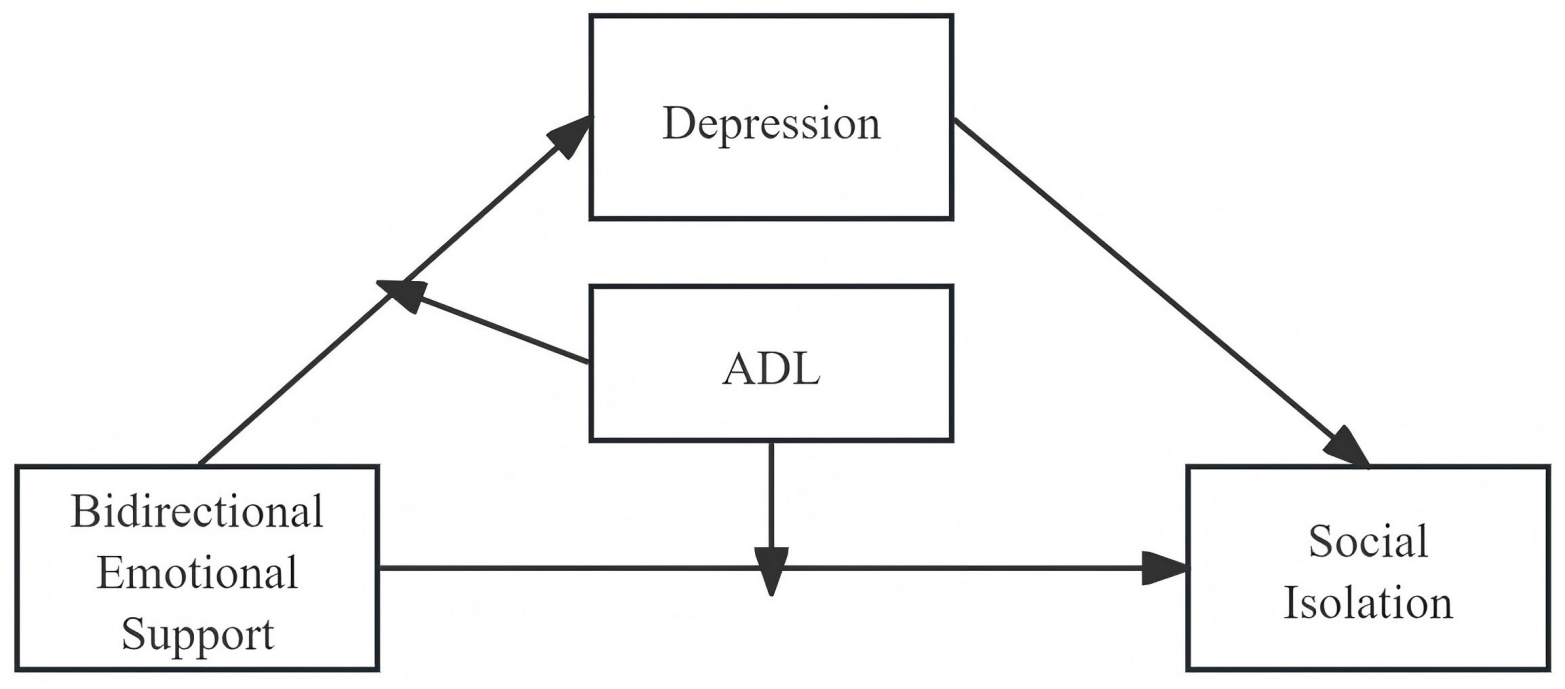
Hypothesis model diagram.

ADL refer to the basic self-care tasks individuals perform daily to maintain independent living in home and community settings, reflecting the physical functioning of older adults (32). It typically encompasses two major categories: basic activities of daily living (BADL) (32) and instrumental activities of daily living (IADL) (33). Research indicates that when older adults experience severe limitations in ADL, the positive effect of emotional support on social participation is diminished, thereby restricting their social networks (34). This suggests that ADL may moderate the pathway through which bidirectional emotional support influences social isolation. Given Kim et al.'s (35) findings that ADL and depression co-vary, older adults with greater ADL impairment are more prone to depression. We hypothesize that baseline physical functioning moderates the association between emotional support and depressive symptoms. Healthy aging is a multifaceted process that includes quality of life, emotional wellbeing, social engagement, and physical health (36). The aging population has heightened the urgency for comprehensive policies and programs, particularly as loneliness increasingly garners attention as a key social determinant of health (37). This study defines “intergenerational emotional support” as bidirectional emotional support, rather than unidirectional giving or receiving, thus addressing a gap in the existing literature. It simultaneously examines both the psychological pathway and the functional pathway within the same model. By setting ADL as a moderating variable, this study addresses the question of “under what physical functional conditions emotional support is most effective,” offering an empirical basis for precision interventions. Additionally, it provides supporting evidence for healthy aging policies, offers guidance for family-centered interventions, and establishes an evidence base for scalable community programs. This study aimed to examine the relationship between bidirectional emotional support, depression, ADL, and social isolation in older adults. In this study, bidirectional emotional support is treated as the independent variable, social isolation as the dependent variable, depression as the mediating variable, and ADL as the moderating variable, testing the following hypotheses:

1. Bidirectional emotional support positively predicts social isolation in older adults;
2. Depression mediates the relationship between bidirectional emotional support and social isolation in older adults;
3. ADL moderates the effect of bidirectional emotional support on social isolation in older adults;
4. ADL moderates the relationship between bidirectional emotional support and depression in older adults. (The hypothetical model in this study is shown in Figure 1).

## 2 Methods

### 2.1 Study design

This cross-sectional study, conducted in accordance with the STROBE statement (38), used a paper-based questionnaire to investigate community-dwelling older adults in China. The study was carried out between July 2022 and November 2022.

### 2.2 Sampling and recruitment

Participants were recruited from community-dwelling older adults in Yaohai District, Hefei City. Community-dwelling older adults were informed about the study in advance through offline community outreach and online WeChat group announcements. Due to the challenges of achieving full population coverage, this sample was limited to community residents in Hefei City, excluding older adults individuals residing in nursing homes. A stratified random sampling method was employed, with communities stratified by urban vs. rural residence. Eight community centers were then randomly selected as survey sites, with four centers in each stratum. This study analyzed a total of 19 variables. Using Kendall's approximate sample size estimation method (which recommends a sample size of at least 10–15 times the number of variables), the minimum required sample size was 190. To account for a 20% non-response rate, a total of 1,150 questionnaires were distributed (39). The inclusion and exclusion criteria are outlined in Table 1.

**Table 1:** Inclusion and exclusion criteria.

**Inclusion criteria**	**Exclusion criteria**
Age ≥60 years	Severe cognitive or psychiatric impairment
Residence in the community for ≥6 months	Significant communication barriers (e.g., visual, auditory, or verbal)
Voluntary participation with informed consent	Refusal to participate or poor compliance
Ability to communicate in Mandarin or the Hefei dialect	

### 2.3 Data collection

Data collection took place between July and November 2022. The research team conducted one-on-one, face-to-face interviews with respondents using two methods: in-home visits and centralized interviews at community activity centers. Respondents provided informed consent, and daily survey data were entered into the database using Epidata 3.1 and cross-checked for accuracy. On-site Questionnaire Verification: missing entries ≤ 20% were completed during follow-up visits. Entries with >20% missing or lacking key variables were considered invalid. A total of 14 questionnaires were excluded, resulting in 1,136 valid responses.

Thirteen investigators were involved in the study: six community nurses and seven graduate students, all of whom received standardized training. The training content includes a detailed explanation of each questionnaire item, two rounds of role-play exercises, and on-site error correction and assessment, with a certification requirement of a score ≥90.

A dual-investigator system was used, with one investigator asking questions and the other recording responses, switching roles daily. During the survey, standardized introductory statements and neutral follow-up questions were employed, prohibiting leading or excessive explanations. All investigators signed confidentiality agreements. Questionnaires were anonymized using identification numbers, and personal information (e.g., names and phone numbers) was removed from the database.

For illiterate or visually impaired individuals, investigators either read the questionnaire aloud or provided large-print answer sheets along with magnifying glasses. Respondents completed the questionnaire independently or authorized investigators to do so on their behalf, ensuring both data quality and ethical compliance.

### 2.4 Measures

#### 2.4.1 General information questionnaire

Based on an extensive review of the literature and consideration of the contextual factors, the researcher developed a general information questionnaire, which included items on gender, age, living arrangement, self-assessment of health, and per capita monthly income.

#### 2.4.2 Social network scale (LSNS-6)

The LSNS-6 was used to assess perceived social isolation in older adults (40). Chang et al. (41). translated the scale into Chinese and validated its reliability and validity among the older population, demonstrating strong reliability and validity. The scale comprises 6 items, utilizing a 6-point Likert scale with a total score range from 0 to 30. It assesses two dimensions: family network and friendship network. Higher scores indicate stronger social networks. A total score of less than 12 indicates insufficient social networks, while a score of 12 or higher indicates good social relationships and social support. The Cronbach's alpha coefficient for this study was 0.776.

#### 2.4.3 Activities of daily living scale (ADL Scale)

The ADL Scale was used to assess ADL in the older adults (42). It is widely used in epidemiological research on older adults in China and has demonstrated good reliability and validity (43). The scale consists of 14 items, utilizing a 4-point Likert scale, with a total score range from 14 to 56. It assesses two core dimensions: BADL and IADL. In this study, it was included as a continuous variable in the analysis, with higher scores indicating more severe impairment in ADL. A score of ≤ 14 represents normal function, while a score >14 indicates functional impairment. In this study, the Cronbach's alpha coefficient for the scale was 0.900.

#### 2.4.4 Geriatric depression scale-15 (GDS-15)

The GDS-15 scale developed by Parmelee and Katz was used to assess depressive symptoms (44). It was applied to older adults Chinese individuals by Tang et al. (45), demonstrating strong reliability and validity. The scale consists of 15 items that assess participants' symptoms over the past week using a binary “yes/no” response format, with a total score range of 0–15. A score of 0–4 indicates no depression, 5–8 indicates mild depression, 9–11 indicates moderate depression, and 12–15 indicates severe depression. In this study, the Cronbach's alpha coefficient for the scale was 0.753.

#### 2.4.5 Intergenerational support scale

The Intergenerational Support Scale, developed by Huang et al. (46), was applied to an older population and demonstrated strong reliability and validity (47). This study focused solely on the bidirectional emotional support dimension of the scale to assess the bidirectional emotional support status among older adults, which includes three questions: (1) closeness of parent-child relationships, (2) harmony between parents and children, and (3) frequency of discussing personal matters between parents and children over the past year. A 3-point Likert scale was used, with total scores ranging from 3 to 9. Higher scores reflect greater levels of intergenerational support. The Cronbach's alpha coefficient for this study was 0.883.

### 2.5 Data analysis

Statistical analysis was conducted using SPSS version 27.0. Harman's one-way test was employed to assess common method bias, and covariance diagnostics were performed. Descriptive statistics were applied to the collected data, with frequency data presented as *n* (%) and continuous data described using appropriate measures. Spearman's correlation analysis was performed with a two-tailed test at an α level of 0.05.

We used PROCESS Model 4 to examine the mediating effect (48). To assess whether the indirect effect was influenced by the moderator variable, we applied Model 8. This model allows the M → Y path to vary with the moderator and provides indices for the moderated mediation effect (49). Since the sampling distribution of mediation effects often deviates from normality, this study uses a bias-corrected non-parametric Bootstrap method to estimate 95% confidence intervals. The sampling process is repeated 5,000 times, balancing computational load with estimation precision, a setting recommended for mediation effect analyses (50). All statistical tests were two-tailed, with significance set at *P* < 0.05.

### 2.6 Ethical considerations

The study received ethical approval from the Medical Ethics Committee of Anhui Medical University (No. 81220209) and adhered to the Declaration of Helsinki. All participants provided informed consent.

## 3 Results

### 3.1 Analysis of common method bias

Exploratory factor analysis of the raw data using Harman's one-way test revealed that nine factors had eigenvalues greater than 1, with the first explaining 21.667% of the total variance (below the 40% threshold), indicating no significant common method bias. Covariance diagnostics for the four variables (social isolation, ADL, depression, and bi-directional emotional support) indicated that the inflation factors were all below 10 (VIF: 1.043–1.090), and the tolerances ranged from greater than 0.1 to less than 1 (Tol: 0.918–0.959), confirming the absence of serious multicollinearity among the variables.

### 3.2 Basic information of the study population

This study surveyed a total of 1,136 community-dwelling older adults, consisting of 449 males (39.5%) and 687 females (60.5%). Of these, 967 (85.1%) were primarily urban residents, while 169 (14.9%) were rural residents. The majority of participants had an educational attainment of primary school or below (874, 76.9%), followed by those with junior high school education (148, 13.0%). The largest group of participants lived with their spouses (476, 41.9%), while 128 (11.3%) lived alone. Further details are provided in Table 2.

**Table 2:** Basic information on the study population.

**Category**	**Subgroup**	***N***	**Percentage (%)**
Gender	Male	449	39.5
	Female	687	60.5
Age (years)	60–69	587	51.7
	70–79	427	37.6
	≥80	122	10.7
Household Registration	Urban	967	85.1
	Rural	169	14.9
Marital status	Married	962	84.7
	Widowed	169	14.9
Educational attainment	Primary school or below	874	76.9
	Junior high school	148	13.0
	High school/vocational	92	8.1
	College or above	22	1.9
Monthly household income (¥)	≤1,000	641	56.4
	1,001–2,000	190	16.7
	2,001–3,000	137	12.1
	3,001–5,000	83	7.3
	≥5,000	85	7.5
Self-rated health	Very good	624	54.9
	Good	275	24.2
	Average	200	17.6
	Poor	37	3.3
Residence type	Urban	967	85.1
	Rural	169	14.9
Social participation	Frequently (≥3 times/week)	541	47.6
	Occasionally (1–2 times/week)	212	18.7
	Rarely or never (≤1 time/week)	383	33.7
Source of income	Pension	203	17.9
	Children's support	366	32.2
	Government subsidies	302	26.6
	Others	265	23.3
Current living situation	Living alone	128	11.3
	Living with spouse	476	41.9
	Living with children	236	20.8
	Living with spouse and children	288	25.4
	Other	8	0.7
Physical exercise	No	220	19.4
	Yes	913	80.6
	Others	265	23.3
Current living situation	Living alone	128	11.3

### 3.3 Descriptive statistics and inter-correlations among study variables

Correlation analysis revealed a significant positive correlation between bidirectional emotional support and social isolation, and a significant negative correlation with depression in older adults. A significant negative correlation was observed between depression and social isolation. ADL exhibited a significant negative correlation with Bidirectional emotional Support and social isolation, and a significant positive correlation with depression (*P* < 0.05). Please refer to Table 3 for further details.

**Table 3:** Descriptive statistics and partial correlation analysis of variables.

**Variable**	**Mean ±SD**	**Depression**	**ADL**	**Social isolation**	**Bidirectional emotional support**
Depression	3.89 ± 2.90	1.000	0.243^**^	−0.305^**^	−0.231^**^
Social isolation	12.07 ± 4.90	−0.305^**^	−0.160^**^	1.000	0.248^**^
Bidirectional emotional support	7.88 ± 1.41	−0.231^**^	−0.056^**^	0.248^**^	1.000
ADL	15.17 ± 3.62	0.243^**^	1.000	−0.160^**^	−0.056^**^

*P* < 0.01 (two-tailed), correlation is significant.

^**^Indicates statistical significance at *P* < 0.01.

### 3.4 Mediating role of depression

The mediating role of depression between bidirectional emotional support and social isolation in older adults was examined using Model 4 in PROCESS 4.0. Bidirectional emotional support was found to positively predict social isolation in older adults (β = 0.213, *t* = 7.437, *P* < 0.001), supporting Hypothesis 1. Additionally, bidirectional emotional support negatively predicted depression (β = −0.255, *t* = −8.852, *P* < 0.001), and depression negatively predicted social isolation in older adults (β = −0.259, *t* = −9.021, *P* < 0.001). The Bootstrap 95% confidence intervals for both the direct effect of bidirectional emotional support on social isolation and the mediating effect of depression did not include zero, indicating that depression partially mediated the relationship between bidirectional emotional support and social isolation, thus supporting Hypothesis 2. The direct effect (0.213) and the mediating effect (0.066) accounted for 76.34% and 23.66%, respectively, of the total effect (0.279). See Tables 4, 5 for further details.

**Table 4:** Regression analyses of bidirectional emotional support with depression and social isolation.

**Dependent variable**	**Predictor variable**	**Model fit indices**			**Regression coefficients**		
		***R***	***R2***	***F***	β **(95%CI)**	***T***	***P-*** **value**
Depression	Bidirectional emotional Support	0.271	0.073	29.552	−0.255(−0.312 to −0.199)	−8.852	0.000
Social isolation	Bidirectional emotional Support	0.379	0.144	46.974	0.213(0.157–0.269)	7.437	0.000
Depression				−0.259(−0.3165 to −0.203)	−9.021	0.000

**Table 5:** Mediation effect test for depression.

**Pathway**	**Effect size**	**Standard error**	**95% CI lower**	**95% CI upper**	**Relative effect size**
Total effect: bidirectional emotional Support → Social Isolation	0.279	0.029	0.223	0.336	
Direct effect: bidirectional emotional Support → Social Isolation	0.213	0.029	0.157	0.269	76.34%
Mediated effect: bidirectional emotional Support → Depression → Social Isolation	0.066	0.011	0.046	0.089	23.66%

### 3.5 Test of the moderating effect of ADL on the mediating effect

**Figure 2:**
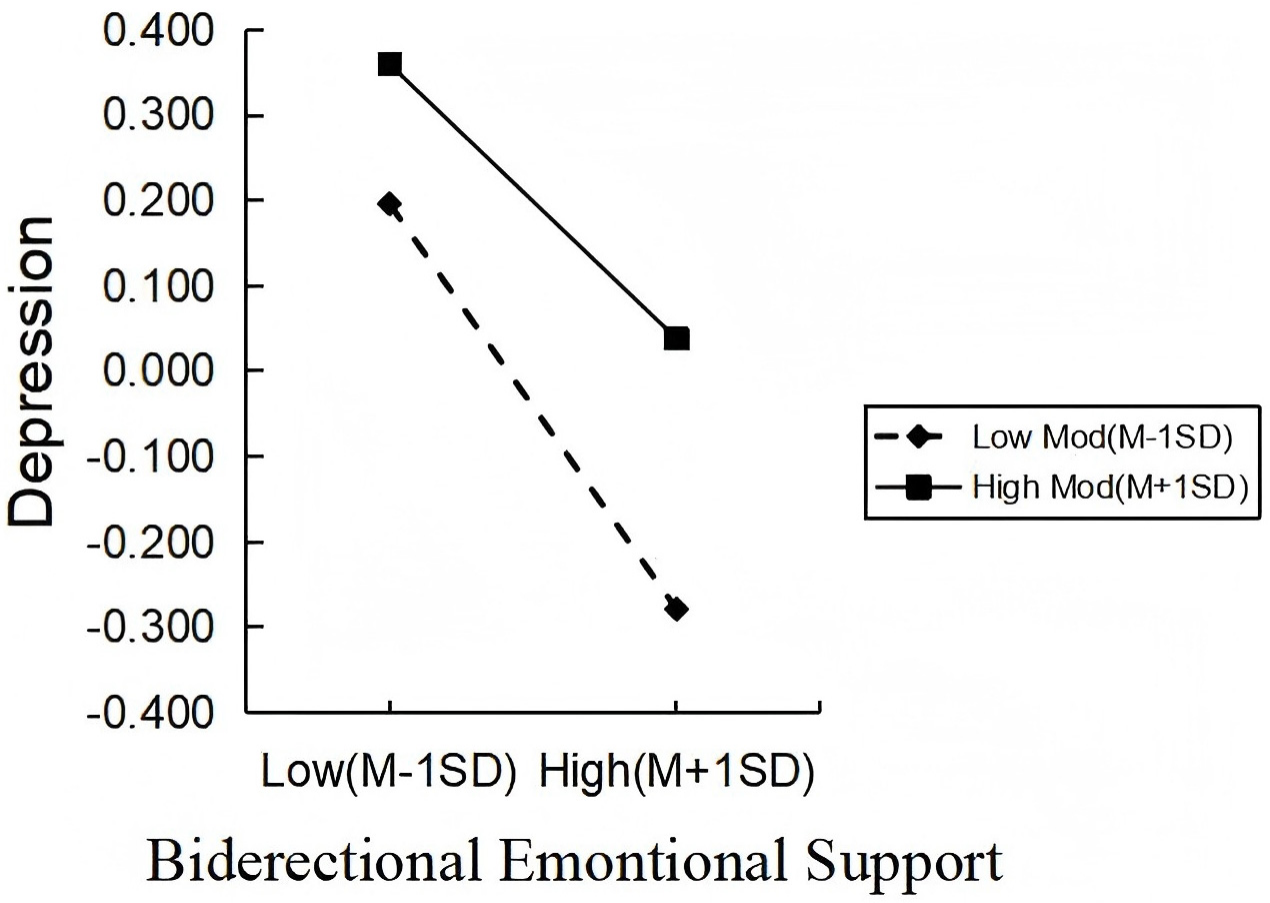
The moderating role of ADL in the effect of bidirectional emotional support on depression.

The moderated mediation model was analyzed using Model 8, with results presented in Table 6. The interaction term between ADL and bidirectional emotional support significantly predicted depression (β = 0.068, *t* = 2.677, *P* < 0.01), indicating a significant moderating effect of ADL; thus, Hypothesis 4 was supported. In contrast, the interaction term between ADL and bidirectional emotional support was not significant in predicting social isolation (β = 0.033, *t* = 1.348, *P* > 0.05), thus Hypothesis 3 was not supported. Further analysis using simple slope techniques revealed that the negative associations between bi-directional emotional support and depression were statistically significant at both low ADL levels (one standard deviation below the mean) and high ADL levels (one standard deviation above the mean; βsimple = −0.264, *t* = −8.837, *P* < 0.001; βsimple = −0.179, *t* = −4.896, *P* < 0.001) see Figure 2. Further analysis of the mediating effect of depression at the three ADL levels revealed that the mediating effect of depression between bi-directional emotional support and social isolation was statistically significant at both low ADL levels and high ADL levels [*r* = 0.066, CI = (0.046, 0.090); *r* = 0.016, CI = (0.016, 0.069)]. See Table 7 for details.

**Table 6:** Tests for the moderating mediating effect of ADL.

**Dependent variable**	**Predictor variable**	**Model fit indices**			**Regression coefficients**		
		***R***	***R2***	***F***	β **(95%CI)**	***T***	***P-*** **value**
Depression	Bidirectional Emotional support	0.314	0.099	24.465	−0.244 (−0.300 to −0.188)		
	ADL				0.200(0.130–0.269)	5.598	0.000
	Bidirectional emotional support × ADL				0.068(0.018–0.117)	2.677	0.008
Social isolation	Bidirectional emotional support	0.391	0.153	33.558	0.205(0.149–0.261)	7.16	0.000
	Depression				−0.249(−0.306 to −0.192)	−8.586	0.000
	ADL				−0.075(−0.144 to −0.006)	−2.143	0.032
	Bidirectional emotional support × ADL				0.033(−0.015 to 0.081)	1.348	0.178

**Table 7:** Indirect effects of depression at different levels of ADL.

**ADL**	**Effect size**	**Bootstrapped standard error**	**95% CI**
M−1SD	0.066	0.011	0.046–0.090
M	0.061	0.011	0.041–0.083
M+1SD	0.045	0.013	0.016–0.069

## 4 Discussion

This study explores the impact of bidirectional emotional support on social isolation, investigating its underlying mechanisms through two dimensions: psychological pathways (depression) and physiological pathways. It addresses a gap in the existing research, as few studies have explicitly examined the buffering effect of bidirectional emotional support on social isolation in older adults. A positive correlation was found between bidirectional emotional support and social isolation. Depression partially mediated the effect of bidirectional emotional support on social isolation, while ADL moderated the indirect pathway from bidirectional emotional support to depression in the mediation model.

This study demonstrated that bidirectional emotional support significantly and positively predicts social networks: the richer the support, the lower the risk of social isolation among older adults. Consistent with Brandt et al. (51), such within-family exchanges provide psychological comfort and help ward off negative emotions. The theory of intergenerational solidarity highlights emotional solidarity as a key dimension that sustains kinship networks (14). When older parents and their children mutually express care and empathy, intergenerational identification is strengthened, conflicts are reduced, and kinship networks grow accordingly. Social support theory further posits a reciprocity effect: children who receive emotional support tend to replicate altruistic behaviors, extending mutual aid to neighbors and friends, which in turn enlarges the older adults' extra-familial networks. By reinforcing intergenerational bonds and spilling over into reciprocal exchanges, bidirectional emotional support simultaneously broadens both kin and non-kin networks, offering a valuable intervention target for reducing social isolation in.

This study revealed that depression partially mediates the link between bidirectional emotional support and social isolation. Specifically, bidirectional emotional support not only directly reduces the risk of social isolation but also indirectly mitigates it by alleviating depressive symptoms. By enhancing older adults' emotional regulation and psychological resilience, bidirectional emotional support lowers the likelihood of depression, consistent with the findings of Wu and Chiou (52). Emotional support provides individuals with channels for expression, understanding, and comfort, helping to alleviate feelings of loneliness and helplessness, thereby reducing depressive symptoms. The alleviation of depressive symptoms further encourages older adults to actively engage in community activities, maintain existing social relationships, and establish new interpersonal connections, thereby expanding their social networks. This aligns with the findings of Luo (53) Conversely, individuals with higher levels of depression often avoid social interactions due to low mood, diminished self-esteem, and reduced social motivation. This leads to decreased social participation, a shrinking social network, and ultimately exacerbates social isolation (29). Thus, bidirectional emotional support mitigates the risk of social isolation among older adults by alleviating depression. Future interventions should focus on improving the quality of bidirectional emotional support while integrating mental health promotion strategies to effectively expand social networks and reduce social isolation.

This study demonstrated that ADL significantly moderates how bidirectional emotional support affects depression. Specifically, among older adults with higher ADL levels, emotional support exerts a stronger alleviating effect on depression, consistent with the findings of Zhou et al. (54) This may occur because individuals with higher ADL have greater mobility, which enables them to more actively translate emotional support into tangible social behaviors, thus effectively alleviating negative emotions. Conversely, those with severely limited ADL face restricted activity spaces and diminished coping resources, making it difficult to release stress through external interactions. As a result, the psychological buffering effect of emotional support is weakened (55, 56). Notably, ADL did not significantly moderate the direct effect of bidirectional emotional support on social isolation. This contrasts with findings from Zhao et al. (57), who reported that higher ADL levels more effectively moderated loneliness among older adults. This discrepancy may be due to the relatively mild ADL impairments observed in most older participants in this study, whose social engagement remained largely unaffected, thereby weakening the moderating effect.

This study presents several important research implications. First, at the theoretical level, it enhances our understanding of the mechanisms of social isolation in aging societies, providing a foundation for future intervention studies aimed at addressing social isolation. Second, at the practical level, “family bidirectional emotional support cultivation” and the “emotional needs fulfillment rate among older adults” should be integrated into the performance evaluation system for older care services. Communities can also organize family communication workshops to strengthen intergenerational reciprocal relationships. Primary healthcare institutions should implement routine depression screening protocols, with a focus on individuals with impaired ADL. High-risk individuals require comprehensive interventions that combine psychological support with personalized ADL training to improve both mental health and functional capacity.

## 5 Limitations

This study has several limitations. First, the geographical scope was limited (only Anhui community). Second, subjective assessment of emotional support, no objective measures. Third, cross-sectional design limiting causal inference. Suggest ways future research could address these limitations: longitudinal studies, multicenter samples, more detailed assessment of emotional support.

## 6 Conclusion

In summary, bidirectional emotional support reduces social isolation in older adults, partially through alleviating depressive symptoms. Maintaining ADL functionality enhances this protective effect, highlighting the importance of integrated family and community interventions to promote mental health and social engagement in aging populations. Future research should focus on longitudinal designs and intervention studies to elucidate the mechanisms involved.

## Data Availability

The raw data supporting the conclusions of this article will be made available by the authors, without undue reservation.

## References

[1] Nicholson NR Jr. Social isolation in older adults: an evolutionary concept analysis. J Adv Nurs. (2009) 65:1342–52. 10.1111/j.1365-2648.2008.04959.x 19291185

[2] Evans IE Llewellyn DJ Matthews FE Woods RT Brayne C Clare L. Social isolation, cognitive reserve, and cognition in healthy older people. PloS ONE. (2018) 13:e0201008. 10.1371/journal.pone.0201008 30118489 PMC6097646

[3] Cudjoe TK Roth DL Szanto SL Wolff JL Boyd CM Thorpe RJ Jr. The epidemiology of social isolation: National health and aging trends study. J Gerontol Series B. (2020) 75:107–13. 10.1093/geronb/gby037 29590462 PMC7179802

[4] Oe N Tadaka E. Differences in loneliness and social isolation among community–dwelling older adults by household type: a Nationwide Survey in Japan. Healthcare. (2023) 11:1647. 10.3390/healthcare11111647 37297787 PMC10252928

[5] Su Y Rao W Li M Caron G D'Arcy C Meng X. Prevalence of loneliness and social isolation among older adults during the COVID-19 pandemic: a systematic review and meta–analysis. Int Psychogeriatr. (2023) 35:229–41. 10.1017/S1041610222000199 35357280

[6] Zlotnick H Hoffman GJ Nuliyalu U Engler TA Langa KM Ryan AM. Is social capital protective against hospital readmissions? BMC Health Serv Res. (2020) 20:248. 10.1186/s12913-020-05092-x 32209077 PMC7092426

[7] Barnes TL Ahuja M MacLeod S Tkatch R Albright L Schaeffer JA. Loneliness, social isolation, and all–cause mortality in a large sample of older adults. J Aging Health. (2022) 34:883–92. 10.1177/08982643221074857 35234547 PMC9483694

[8] Cudjoe TKM Prichett L Szanton SL Roberts Lavigne LC Thorpe RJ Jr. Social isolation, homebound status, and race among older adults: findings from the National Health and Aging Trends Study (2011–2019). J Am Geriatr Soc. (2022) 70:2093–100. 10.1111/jgs.17795 35415872 PMC9283207

[9] Wang S Molassiotis A Guo C Leung ISH Leung AYM. Association between social integration and risk of dementia: a systematic review and meta–analysis of longitudinal studies. J Am Geriatr Soc. (2023) 71:632–45. 10.1111/jgs.18094 36307921

[10] Chamberlain SA Bronskill SE Hsu Z Youngson E Gruneir A. Resident loneliness, social isolation and unplanned emergency department visits from supportive living facilities: a population–based study in Alberta, Canada. BMC Geriatr. (2022) 22:21. 10.1186/s12877-021-02718-5 34979960 PMC8725434

[11] Elovainio M Hakulinen C Pulkki–Råback L Virtanen M Josefsson K Jokela M. Contribution of risk factors to excess mortality in isolated and lonely individuals: an analysis of data from the UK Biobank cohort study. Lancet Public Health. (2017) 2:e260–6. 10.1016/S2468-2667(17)30075-0 28626828 PMC5463031

[12] Fakoya OA McCorry NK Donnelly M. Loneliness and social isolation interventions for older adults: a scoping review of reviews. BMC Public Health. (2020) 20:129. 10.1186/s12889-020-8251-6 32054474 PMC7020371

[13] Wang F Gao Y Han Z Yu Y Long Z Jiang X. A systematic review and meta–analysis of 90 cohort studies of social isolation, loneliness and mortality. Nat Human Behav. (2023) 7:1307–19. 10.1038/s41562-023-01617-6 37337095

[14] Bengtson VL Roberts RE. Intergenerational solidarity in aging families: an example of formal theory construction. J Marriage Fam. (1991) 53:856–70. 10.2307/352993

[15] Melchiorre MG Socci M Lamura G Quattrini S. Perceived loneliness, social isolation, and social support resources of frail older people ageing in place alone in Italy. Healthcare. (2024) 12:875. 10.3390/healthcare12090875 38727432 PMC11083615

[16] Gilligan M Suitor JJ Rurka M Silverstein M. Multigenerational social support in the face of the COVID−19 pandemic. J Fam Theory Rev. (2020) 12:431–47. 10.1111/jftr.12397 34367339 PMC8340915

[17] Maton KI. Social support, organizational characteristics, psychological well-being, and group appraisal in three self-help group populations. Am J Commun Psychol. (1988) 16:53–77. 10.1007/BF00906072 3369383

[18] Antonucci TC Lansford JE Schaberg L Smith J Baltes M Akiyama H. Widowhood and illness: a comparison of social network characteristics in France, Germany, Japan, and the United States. Psychol Aging. (2001) 16:655. 10.1037//0882-7974.16.4.655 11766919

[19] Dong Y Cheng L Cao H. Impact of informal social support on the mental health of older adults. Front Public Health. (2024) 12:1446246. 10.3389/fpubh.2024.1446246 39391160 PMC11464432

[20] Ren K Lan J Ge L Zhou L. The impact of intergenerational support on the mental health of older adults: a discussion of three dimensions of support. Front Public Health. (2025) 13:1467463. 10.3389/fpubh.2025.1467463 40313498 PMC12043444

[21] Li C Jiang S Zhang X. Intergenerational relationship, family social support, and depression among Chinese elderly: a structural equation modeling analysis. J Affect Disord. (2019) 248:73–80. 10.1016/j.jad.2019.01.032 30716614

[22] Jiang Z Liu H Deng J Ye Y Li D. Influence of intergenerational support on the mental health of older people in China. PloS ONE. (2024) 19:e0299986. 10.1371/journal.pone.0299986 38635847 PMC11025908

[23] Li W Zhao N Yan X Zou S Wang H Li Y. The prevalence of depressive and anxiety symptoms and their associations with quality of life among clinically stable older patients with psychiatric disorders during the COVID-19 pandemic. Transl Psychiatry. (2021) 11:75. 10.1038/s41398-021-01196-y 33500389 PMC7835649

[24] Avasthi A Grover S. Clinical practice guidelines for management of depression in elderly. Indian J Psychiatry. (2018) 60(Suppl 3):S341–62. 10.4103/0019-5545.224474 29535469 PMC5840909

[25] Olabisi OI Faronbi J Adedeji P Ademuyiwa G Gambari Y Lasisi A. Influence of family and friends level of social support on psychological symptoms among the older adults in Nigeria. SAGE Open Nurs. (2023) 9:23779608231187778. 10.1177/23779608231187778 37476332 PMC10354820

[26] Uchino BN Cacioppo JT Kiecolt–Glaser JK. The relationship between social support and physiological processes: a review with emphasis on underlying mechanisms and implications for health. Psychol Bull. (1996) 119:488–531. 10.1037//0033-2909.119.3.488 8668748

[27] Heller K Thompson MG Vlachos–Weber I Steffen AM Trueba PE. Support interventions for older adults: confidante relationships, perceived family support, and meaningful role activity. Am J Community Psychol. (1991) 19:139–46. 10.1007/BF00942262 1867147

[28] Sun Q Wang Y Lu N Lyu S. Intergenerational support and depressive symptoms among older adults in rural China: the moderating roles of age, living alone, and chronic diseases. BMC Geriatr. (2022) 22:83. 10.1186/s12877-021-02738-1 35086485 PMC8796626

[29] Santini ZI Jose PE York Cornwell E Koyanagi A Nielsen L Hinrichsen C. Social disconnectedness, perceived isolation, and symptoms of depression and anxiety among older Americans (NSHAP): a longitudinal mediation analysis. Lancet Public Health. (2020) 5:e62–70. 10.1016/S2468-2667(19)30230-0 31910981

[30] Wang S Lin J Kuang L Yang X Yu B Cui Y. Risk factors for social isolation in older adults: a systematic review and meta–analysis. Public Health Nurs. (2024) 41:200–8. 10.1111/phn.13266 38037451

[31] Jahangir S Patil DS Gangopadhyay J Vogt TC. Understanding intergenerational dynamics and social support's impact on health and well–being of older adults in South Asia: a scoping review. Syst Rev. (2025) 14:86. 10.1186/s13643-025-02833-z 40217304 PMC11987332

[32] Lesher DA.–M, Mulcahey M, Hershey P, Stanton DB, Tiedgen AC. Alignment of outcome instruments used in hand therapy with the occupational therapy practice framework: domain and process and the international classification of functioning, disability and health: a scoping review. Am J Occup Ther. (2017) 71:7101190060p1–12. 10.5014/ajot.2017.016741 28027048

[33] Depp CA Jeste DV. Definitions and predictors of successful aging: a comprehensive review of larger quantitative studies. Am J Geriatr Psychiatry. (2006) 14:6–20. 10.1097/01.JGP.0000192501.03069.bc 16407577

[34] Tomioka K Kurumatani N Hosoi H. Association between social participation and instrumental activities of daily living among community–dwelling older adults. J Epidemiol. (2016) 26:553–61. 10.2188/jea.JE20150253 27180933 PMC5037253

[35] Kim BJ Liu L Nakaoka S Jang S Browne C. Depression among older Japanese Americans: the impact of functional (ADL & IADL) and cognitive status. Soc Work Health Care. (2018) 57:109–25. 10.1080/00981389.2017.1397588 29236614

[36] Sanchís–Soler G Sebastiá–Amat S Parra–Rizo MA. Mental health and social integration in active older adults according to the type of sport practiced. Acta Psychol. (2025) 255:104920. 10.1016/j.actpsy.2025.104920 40154052

[37] Parra–Rizo MA Cigarroa I Pereira C Hernández–Sánchez S González–Moreno J. Abordando la soledad en la formación de profesionales sanitarios y en la práctica clínica. Educ Méd. (2025) 26:101050. 10.1016/j.edumed.2025.101050

[38] Von Elm E Altman DG Egger M Pocock SJ Gøtzsche PC Vandenbroucke JP. The Strengthening the Reporting of Observational Studies in Epidemiology (STROBE) statement: guidelines for reporting observational studies. Lancet. (2007) 370:1453–7. 10.1016/S0140-6736(07)61602-X 18064739

[39] Preacher KJ Kelley K. Effect size measures for mediation models: quantitative strategies for communicating indirect effects. Psychol Methods. (2011) 16:93. 10.1037/a0022658 21500915

[40] Lubben J Blozik E Gillmann G Iliffe S von Renteln Kruse W Beck JC. Performance of an abbreviated version of the Lubben Social Network Scale among three European community–dwelling older adult populations. Gerontologist. (2006) 46:503–13. 10.1093/geront/46.4.503 16921004

[41] Chang Q Sha F Chan CH Yip PS. Validation of an abbreviated version of the Lubben Social Network Scale (“LSNS−6”) and its associations with suicidality among older adults in China. PloS ONE. (2018) 13:e0201612. 10.1371/journal.pone.0201612 30071067 PMC6072030

[42] Katz S. Assessing self–maintenance: activities of daily living, mobility, and instrumental activities of daily living. J Am Geriatr Soc. (1983) 31:721–7. 10.1111/j.1532-5415.1983.tb03391.x 6418786

[43] Zhang Y Xiong Y Yu Q Shen S Chen L Lei X. The activity of daily living (ADL) subgroups and health impairment among Chinese elderly: a latent profile analysis. BMC Geriatr. (2021) 21:30. 10.1186/s12877-020-01986-x 33413125 PMC7791986

[44] Parmelee PA Katz IR. Geriatric depression scale. J Am Geriatr Soc. (1990) 38:1379. 10.1111/j.1532-5415.1990.tb03461.x 2254577

[45] Tang D. Application of short form geriatric depression scale (GDS−15) in Chinese elderly. Chin J Clin Psychol. (2013) 21:402–5.32663994

[46] Huang Q Du P Chen G. The intergenerational support between adult children and older adults and its associated factors. Popul Dev. (2018) 24:20–8.

[47] Liu H Ma Y Lin L Sun Z Li Z Jiang X. Association between activities of daily living and depressive symptoms among older adults in China: evidence from the CHARLS. Front Public Health. (2023) 11:1249208. 10.3389/fpubh.2023.1249208 38035294 PMC10687586

[48] Hayes AF. Introduction to Mediation, Moderation, and Conditional Process Analysis: A Regression–Based Approach. New York, NY: Guilford Publications (2017).

[49] Hayes AF. An index and test of linear moderated mediation. Multivariate Behav Res. (2015) 50:1–22. 10.1080/00273171.2014.962683 26609740

[50] Shrout PE Bolger N. Mediation in experimental and nonexperimental studies: new procedures and recommendations. Psychol Methods. (2002) 7:422. 10.1037//1082-989X.7.4.422 12530702

[51] Brandt L Liu S Heim C Heinz A. The effects of social isolation stress and discrimination on mental health. Transl Psychiatry. (2022) 12:398. 10.1038/s41398-022-02178-4 36130935 PMC9490697

[52] Wu HY Chiou AF. Social media usage, social support, intergenerational relationships, and depressive symptoms among older adults. Geriatr Nurs. (2020) 41:615–21. 10.1016/j.gerinurse.2020.03.016 32268948

[53] Luo M. Social isolation, loneliness, and depressive symptoms: a twelve–year population study of temporal dynamics. J Gerontol B Psychol Sci Soc Sci. (2023) 78:280–90. 10.1093/geronb/gbac174 36315577 PMC9938924

[54] Zhou L Wang W Ma X. The bidirectional association between the disability in activities of daily living and depression: a longitudinal study in Chinese middle–aged and older adults. BMC Public Health. (2024) 24:1884. 10.1186/s12889-024-19421-w 39010036 PMC11247890

[55] Feng Z Li Q Zhou L Chen Z Yin W. The relationship between depressive symptoms and activity of daily living disability among the elderly: results from the China Health and Retirement Longitudinal Study (CHARLS). Public Health. (2021) 198:75–81. 10.1016/j.puhe.2021.06.023 34365109

[56] Nakamura T Michikawa T Imamura H Takebayashi T Nishiwaki Y. Relationship between depressive symptoms and activity of daily living dependence in older Japanese: the Kurabuchi Study. J Am Geriatr Soc. (2017) 65:2639–45. 10.1111/jgs.15107 28952148

[57] Zhao Y Huo X Du H Lai X Li Z Zhang Z. Moderating effect of instrumental activities of daily living on the relationship between loneliness and depression in people with cognitive frailty. BMC Geriatr. (2025) 25:121. 10.1186/s12877-025-05700-7 39984846 PMC11846412

